# TNF-alpha inhibitor adalimumab attenuates endotoxin induced cardiac damage in rats ^1^

**DOI:** 10.1590/s0102-865020200020000002

**Published:** 2020-04-03

**Authors:** Selim Durmaz, Tünay Kurtoğlu, Emin Barbarus, Nükhet Eliyatkın, Mustafa Yılmaz

**Affiliations:** I MD, Aydın Adnan Menderes University, Faculty of Medicine, Department of Cardiovascular Surgery, Aydın, Turkey. Conception and design of the study, acquisition and analysis of data, final approval.; II Associate Professor, Aydın Adnan Menderes University, Faculty of Medicine, Department of Cardiovascular Surgery, Aydın, Turkey. Conception and design of the study, acquisition and analysis of data, final approval.; III Associate Professor, Aydın Adnan Menderes University, Faculty of Medicine, Department of Medical Pathology, Aydın, Turkey. Design of the study, acquisition and analysis of data, final approval.; IV MD, Department of Medical Biochemistry, Faculty of Medicine, Adnan Menderes University, Aydın, Turkey. Design of the study, acquisition and analysis of data, final approval.

**Keywords:** Lipopolysaccharides, Adalimumab, Cytokines, Rats

## Abstract

**Purpose:**

To investigate the effects of adalimumab pretreatment on the lipopolysaccharide-mediated myocardial injury.

**Methods:**

Twenty-eight Wistar rats were randomized into four groups (n=7). Control (C) group animals were injected once a day with intraperitoneal (i.p) 0.9 % saline for two days. In the Adalimumab (Ada) group, adalimumab was injected at a dose of 10 mg/kg/ day (i.p) for two days. Lipopolysaccharide (Lps) group rats were injected with a dose of 5 mg/kg (i.p) lipopolysaccharide. Lipopolysaccharide + Adalimumab (Lps+Ada) group rats received adalimumab before the administration of lipopolysaccharide. The animals were sacrificed 24 h after the last injection and blood samples were obtained for determination of biochemical cardiac injury markers and circulating levels of TNF-α and interleukin-6 (IL-6). Hearts were harvested for histological examination.

**Results:**

Endotoxin exposure resulted in significant increases in serum cardiac injury markers, serum cytokines and histological myocardial injury scores in the Lps group. The levels of circulating cytokines, cardiac injury markers and histological injury scores for myocardial necrosis, perivascular cell infiltration, and inflammation were significantly reduced in Lps+Ada as compared to Lps group (p<0.05).

**Conclusions:**

Adalimumab pretreatment reduces endotoxin-induced myocardial damage in rats. This beneficial effect is thought to be related to the reduction of cytokine release.

## Introduction

Systemic inflammatory response syndrome is a phenomenon in which various stimuli- such as severe trauma, sepsis, acute mesenteric ischemia and major surgery- induce a widespread activation of inflammatory cascades leading to multi-organ failure^1^ . Systemic inflammation may be triggered by lipopolysaccharide (Lps) components of gram negative bacterial wall which are commonly known as endotoxins^2^ . Human intestinal flora produces a considerable amount of endotoxins but the endothelial cell lining in intestines restrict the transfer of these endotoxins into circulation and prevent the systemic inflammation in normal conditions. In the course of major surgical procedures including cardiac surgery with cardiopulmonary bypass and aortic aneurysm repair, the disruption of endothelial cell barrier may permit the passage of endotoxins through the mucosal epithelium to the adjacent tissues or distal organs and provoke a systemic inflammatory response leading to increased postoperative complications^3^ .

The heart is among the most affected organs by the systemic inflammatory response^7^ . Cytokines have been suggested to be responsible for endotoxin-mediated myocardial injury^8 , 9^ . Increased myocardial and serum levels of TNF-α and IL-6 during Lps-mediated systemic inflammatory responses have shown to be associated with both cellular and functional cardiac deterioration^10 , 11^ . Furthermore, inhibition of TNF-α has been observed to attenuate endotoxin induced myocardial injury^12 , 13^ .

Adalimumab is a recombinant human monoclonal anti-TNF-α antibody that blocks the interaction of TNF-α with the cell surface receptors^14^ . It is used in various chronic immune-mediated diseases such as rheumatoid arthritis, inflammatory bowel disease, psoriatic arthritis, vasculitis or ankylosing spondylitis to modify the inflammatory responses^15^ . However, the effect of adalimumab in cytokine-induced inflammatory myocardial damage has not been previously studied. The aim of our experimental study is to investigate the effects of systemic adalimumab treatment on circulating cytokines and myocardial injury in Lps-mediated systemic inflammation.

## Methods

The animal care was conducted in accordance with the National Institute of Health’s Guide for the Care and Use of Laboratory Animals. The animals used in the experiment were obtained from the Aydın Adnan Menderes University Experimental Animals Research and Production Laboratory. Adult female Wistar rats (170-270g) were fed a standard rat chow diet and water ad libitum and kept in cages in a temperature (22°C ± 2°C) and humidity (45-50%) controlled room with a 12-h dark-light cycle and acclimatized for a week before the study. The experimental design and protocol were approved by the Animal Care Committee of Adnan Menderes University (approval number: 64583101/2018/81). The study was performed in the Adnan Menderes University Faculty of Medicine, Experimental Animals Laboratory, Aydın, Turkey. A total of 28 animals were randomly allocated into one of the four following groups each consisting of seven animals: Control (C), Adalimumab (Ada), Lipopolysaccharide (Lps), Lipopolysaccharide + Adalimumab (Lps+Ada) groups.

### Study protocol

***Control (C) Group* (n=7):** Animals were injected once a day with intraperitoneal (i.p) 0.9% saline for two days. After the injection, they were returned to cages and allowed standard rat chow and water ad libitum for 24 h.

***Adalimumab (Ada) Group* (n=7):** Adalimumab (Humira^©^, Vetter Pharma-Fertigung GmBH&Co.KG, Ravensburg/Germany) was injected at a dose of 10 mg / kg / day (i.p) for two days. The animals were returned to cages and allowed standard rat chow and water ad libitum for 24 h after last injection.

***Lipopolysaccharide (Lps) Group* (n=7):** Animals were injected with lipopolysaccharide (LPS) (Escherichia coli 0111:B4 Sigma, Deisenhofen, Germany), with a dose of 5 mg/kg (i.p) then returned to their cages and were allowed standard rat chow and water ad libitum for 24 h^16 , 17^ .

***Lipopolysaccharide + Adalimumab (Lps+Ada) Group* (n=7):** Animals were given Adalimumab treatment as described above before the injection of lipopolysaccharide. Then, they were returned to cages and allowed standard rat chow and water ad libitum for 24 h after the last injection.

### Blood and myocardial tissue sampling

In each group, 24 hours after the last injection animals were anesthetized with a combination of ketamine hydrochloride and xylazine injection (i.p). Then, a median sternotomy was performed and 1 cc of blood was sampled through right atrium puncture via 30 G x 8 mm cannula. After blood sampling, the rats were sacrificed by cervical dislocation and immediately after that, the heart was dissected free from the surrounding tissues, harvested for histopathological examination and fixed in 10% phosphate-buffered formalin. Blood samples were centrifuged at 1000g for 10 min to obtain the serum and were stored at – 80 C^0^ until analysis of serum cytokines.

### Biochemical analysis

Serum Troponin I and creatine kinase muscle-brain (CK-MB) analysis was made by Chemiluminescent micro particle immunoassay (CMIA) method with Autoanalyser (C8000 Architect, Abbott, Abbott Park, IL, U.S.A.) on the same day of sampling^18^ .

The serum concentrations of IL-6 and TNF-α were measured by performing enzyme-linked immunosorbent assay (ELISA) analyses. Inflammatory mediators from each sample were quantified using rat specific ELISA kits (E-EL-R0019, Elabscience Biotechnology Co. WuHan, PRC). Results were calculated using ELISA micro plate reader (DAR 800, Diagnostic Automation, CA 91302, USA) using standard curves and expressed as picograms per milliliter (pg/mL) of serum.

### Histopathologic evaluation

Tissue samples were embedded in paraffin blocks and 4-µm thick sections were obtained across the ventricles. The sections were stained with hematoxylin-eosin and examined under light microscope (Leica DM 1000, Germany) by a blinded pathologist. Myocardial tissue samples were evaluated in terms of congestion, inflammation, necrosis, and perivascular cell infiltration. Histopathological changes were scored on a 4-point semi quantitative scale as follows: none (0), mild (1), moderate (2), severe (3).

### Statistical analysis

Research data were evaluated using the SPSS 24.0 statistical program (IBM Corp., Armonk, NY, USA). The Kolmogorov-Smirnov test was used to evaluate whether the distribution of continuous variables were normal. As continuous variables were normally distributed, descriptive analyses were presented as means ± standard deviations. One-way ANOVA was used to compare parameters among study groups. Levene’s test was used to assess the homogeneity of variances. Post-hoc Tukey test was performed to determine the significance of pairwise differences using the Bonferroni correction to adjust for multiple comparisons. A 5% type-1 error level was used to infer statistical significance.

## Results

### Cardiac biochemical injury markers

Levels of biochemical cardiac injury markers among study groups are demonstrated in Table 1 . The serum levels of Troponin I and CK-MB in Lps group were found to be significantly elevated in comparison with the remaining groups (p<0.05). There were no significant differences regarding the serum levels of biochemical cardiac injury markers among control, Ada and Lps+Ada groups (p>0.05).

**Table 1:** Results of cardiac biochemical injury markers.

Groups	Control (C)	Adalimumab (Ada)	Lipopolysaccharide (Lps)	Lipopolysaccharide + Adalimumab (Lps+Ada)
Troponin I	168.1 ± 89.8	441.6 ± 350.9	2916.7 ± 1184.6^*^	997.1 ± 174.2
CK-MB	0.73 ± 0.1	0.84 ± 0.4	1.53 ± 0.4^†^	0.90 ± 0.4

Data are presented as mean ± standard error of mean. CK-MB: Creatine kinase muscle brain.

P<0.05 was considered as statistically significant.

^*^P<0.05 compared with C, Ada and Lps + Ada groups.

^†^P<0.05 compared with C, Ada and Lps + Ada groups.

### Serum cytokine levels

Serum cytokine levels in study groups are demonstrated in Table 2 . TNF-α and IL-6 serum levels were significantly increased in Lps group as compared to the other study groups (p<0.05). Although, the serum cytokine levels in Ada group were lower than the levels in control group, the differences were not statistically significant (p>0.05).

**Table 2:** - Results of serum cytokines levels.

Groups	Control (C)	Adalimumab (Ada)	Lipopolysaccharide (Lps)	Lipopolysaccharide + Adalimumab (Lps+Ada)
TNF-α	862.4 ± 223.2	726.1 ± 137.1	1189.0 ± 168.8^‡^	831.1 ± 122,4.
IL-6	148.1 ± 30.7	138.4 ± 18.2	232.0 ± 37.6^∫^	174.8 ± 25.7

Data are presented as mean ± standard error of mean. TNF-α: Tumor necrosis factor alpha. IL-6: Interleukin-6.

P<0.05 was considered as statistically significant.

^‡^P<0.05 compared with C, Ada and Lps + Ada groups.

^∫^P<0.05 compared with C, Ada and Lps + Ada groups.

### Histologic evaluation

**Figure 1:**
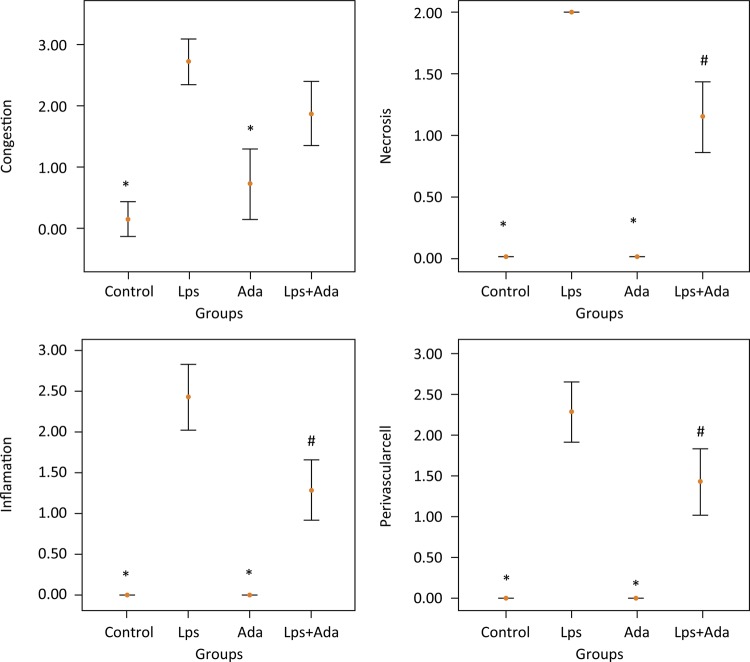
Results of histopathologic injury scores. *p < 0.05 compared with Lps and Lps+Ada groups; #p < 0.05 compared with Lps group.

**Figure 2:**
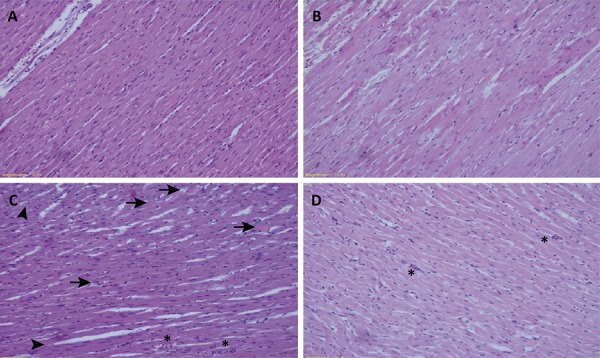
Representative histological samples from groups. Myocardial tissue samples. (A) Control group; normal histological appearance. (B) Adalimumab group; normal histological appearance. (C) Lipopolysaccharide group; diffuse and severe injury: *arrows* indicate inflammation and congestion, *stars* indicate perivenular inflammation and *arrowheads* indicate necrosis. (D) Lipopolysaccharide + Adalimumab group; moderate injury: *stars* indicate perivenular inflammation.

The results of histopathological injury scores are shown in Figure 1 . Histopathologic injury scores in the control and Ada groups were significantly lower than the scores in the Lps and Lps+Ada groups (p<0.05). On the other hand, there were no significant differences between the control and Ada groups in terms of histopathological injury scores (P>0.05). There was no significant difference in terms of congestion among the Lps and Lps+Ada groups (p>0.05). However, necrosis, perivascular cell infiltration and inflammation scores in the Lps + Ada group were significantly lower than the scores in the Lps group (p<0.05). Representative myocardial tissue histologic sections of study groups are sequentially presented in Figure 2 .

## Discussion

In the present study, we used a model of lipopolysaccharide-induced experimental myocardial injury. Our findings indicate that adalimumab treatment significantly reduces circulating cytokines (TNF-α and IL-6), which are released as a response to lipopolysaccharide administration in rats. In animals that are subjected to lipopolysaccharide, adalimumab treatment resulted in a significant reduction in serum levels of cardiac injury markers and amelioration of histopathologic signs of myocardial injury. These findings suggest that TNF- α blocker adalimumab attenuates endotoxin-induced myocardial injury by suppression of cytokine release into the circulation. Furthermore, we also observed that the administration of adalimumab at the selected dose in rats did not cause any cardiac injury whilst slightly reducing the levels of circulating cytokines.

Tumor necrosis factor-alpha is a multifunctional cytokine that has pleiotropic effects in the cardiovascular system. It is primarily produced by lymphocytes and macrophages; however, various other cell types such as cardiomyocytes, resident cardiac macrophages and vascular smooth muscle cells are also capable of TNF-α production^19^ . It acts as a mediator in local homeostasis by regulating cellular growth, cytoprotection, and innate immunity when produced as a part of a physiologic adaptive response. On the other hand; TNF-α plays a crucial role in various cardiovascular pathologies such as atherosclerosis, myocarditis and heart failure as a key molecule of the inflammation^15 , 20^ . It is postulated that an uncontrolled increase of TNF-α production or sustained accumulation of TNF-α leads to detrimental effects on the myocardium. These effects include the induction of apoptosis, enhancement of cytokine production, accumulation of inflammatory cells and decrement of myocardial contractility as a consequence of the deterioration in myocardial calcium metabolism^20^ . Therefore; blockage of TNF-α has been proposed to be a preventive or alleviating therapeutic target particularly in cardiac pathologies in which inflammation plays a role^21^ .

In experimental and clinical conditions, endotoxin-mediated myocardial injury has been demonstrated to be related to increased cytokines^3 , 9 , 22^ . The major cytokines that induce cardiac damage during endotoxemia are shown to include TNF-α and IL-6^10 , 11^ . Xianchu et al. demonstrated that in mice, myocardial TNF-α and IL-6 levels are increased in lipopolysaccharide-mediated myocardial damage and suppression of these cytokines using rutin extract reduces the inflammatory response and damage^12^ . In another experimental study, Chung et al. reported that estrogen causes cardiovascular protective effects via inhibition of TNF-α and IL-6 release in a lipopolysaccharide-induced sepsis model in rats^23^ . Similarly, our findings in the present study indicate that lipopolysaccharide administration results in myocardial damage, which is associated with a significant increment in circulating TNF-α and IL-6 levels in rats. We observed that adalimumab treatment before the administration of lipopolysaccharide causes attenuation of myocardial damage as determined by a significant reduction in cardiac injury markers and the severity of the histopathologic injury.

Adalimumab binds with the soluble and trans-membrane TNF- α molecules with high affinity without binding to the other cytokines^24^ . It has a beneficial disease-modifying effect in various chronic inflammatory pathologies by blocking the activity of TNF-α; however, its effect on serum TNF-α levels is inconsistent. Adalimumab treatment was not observed to cause a significant change in serum TNF-α profile in some clinical studies^25 , 26^ . On the other hand, it was reported to result in a significant decrement in circulating IL-6 levels in several inflammatory diseases^25 , 26^ . The reason for these findings may be the differences in individual genetic features and immunological processes affecting the response to the drug^27 , 28^ .

Although adalimumab has been reported not to bind with rat TNF-α in vitro, various in-vivo experimental studies have established that it can significantly inhibit the overproduction of TNF-α in rats^29^ . It has been shown that adalimumab treatment reduces serum TNF–α levels in diabetic rats and leads to a decrement in both TNF–α and IL-6 serum levels in obese non-diabetic rats^30^ . In addition to this, Cure *et al* .^31^ reported that adalimumab decreases renal tissue cytokine levels and renal damage in a rat model of aortic clamping induced ischemia-reperfusion injury. Adalimumab has also been demonstrated to attenuate the ischemia-reperfusion mediated lung injury through inhibition of the increase in tissue TNF-α levels in rats^32^ . Furthermore, in an experimental setting of non-alcoholic steatohepatitis in rats, adalimumab has been declared to significantly decrease the serum TNF–α and IL-6 levels^33^ . In our experimental model, we found that the increment in levels of TNF–α and IL-6 in circulation as a response to lipopolysaccharide administration is significantly suppressed in adalimumab treated rats. We suggest that the myocardial protective effect of adalimumab, which we observed in this study, is the result of the reduction in circulating cytokines; however, this effect might also be related to the blockage of TNF–α in cardiac tissue.

Adalimumab has been usually used at a single intraperitoneal (i.p) dose of 50 mg/kg in experimental studies in rats, but in clinical practice, it is often delivered as a 40 mg single bolus dose via subcutaneous injection in adults^24 , 30 , 34^ . According to the product information provided by the manufacturer, the highest dose of adalimumab that has been evaluated during clinical trials is multiple intravenous doses of 10 mg/kg^29^ . Based on this knowledge, we preferred to use a dose of 10 mg /kg/day (i.p) and we have repeated the dose for two consecutive days, because the drug absorption and distribution following a single dose are relatively slow in the clinical setting^24^ . In order to determine the effect of the selected dose of adalimumab on serum cytokine profile and myocardium, we have administered the drug without endotoxin challenge in the Ada group. We did not observe any biochemical or histological evidence of myocardial damage associated with adalimumab treatment in our study. Although there was a slight decrease in the levels of serum cytokines in the adalimumab treated rats, the differences were not statistically significant as compared with the control group animals.

There are some limitations of our study; firstly, although we aimed to mimic the production of TNF-α resulting from enhanced bowel permeability and bacterial translocation, which occurs during major surgical procedures, our experimental model of lipopolysaccharide injection does not fully meet the clinical situation. Secondly, we focused mainly on the circulating TNF-α and IL-6 levels but we have not studied the levels of or gene expressions of these cytokines in the myocardium and the levels of other inflammatory mediators that are likely to play a role in myocardial injury. Thirdly, we have not determined the changes in functional cardiac status by means of specific methods such as echocardiography. Therefore, the results of the present study should be interpreted with caution.

## Conclusions

The results of this study showed that adalimumab pretreatment reduces endotoxin-induced myocardial damage in rats. The beneficial effect of adalimumab observed in this study is thought to be related to the reduction of cytokine release. However, further investigation is needed to determine the effect of adalimumab in acute inflammatory conditions that damage the heart and enlighten the possible cardio protective role of adalimumab.

## Footnotes

1 Research performed in Adnan Menderes University Faculty of Medicine, Experimental Animals Laboratory, Aydın, Turkey.
